# Metastatic lymph node ratio can further stratify risk for mortality in medullary thyroid cancer patients: A population-based analysis

**DOI:** 10.18632/oncotarget.11725

**Published:** 2016-08-31

**Authors:** Ning Qu, Rong-liang Shi, Zhong-wu Lu, Tian Liao, Duo Wen, Guo-hua Sun, Duan-shu Li, Qing-hai Ji

**Affiliations:** ^1^ Department of Head and Neck Surgery, Fudan University Shanghai Cancer Center; Department of Oncology, Shanghai Medical College, Fudan University, Shanghai 200032, China; ^2^ Department of General Surgery, Minhang Hospital, Fudan University, Shanghai 201199, China

**Keywords:** medullary thyroid cancer, lymph node dissection, ratio, SEER, survival analysis

## Abstract

Medullary thyroid cancer (MTC) has a propensity to cervical lymph node metastases (LNM). Recent studies have shown that both the number of involved lymph nodes (LNs) and the metastatic lymph node ratio (MLNR) confer prognostic information. This study was to determine the predictive value of MLNR on cancer-specific survival (CSS) in SEER (Surveillance, Epidemiology and End Results)-registered MTC patients treated with thyroidectomy and lymphadenectomy between 1991 and 2012, investigate the cutoff points for MLNR in stratifying risk of mortality and provide evidence for selection of appropriate treatment strategies. X-tile program determined 0.5 as optimal cut-off value for MLNR in terms of CSS in 890 MTC patients. According to multivariate Cox regression analysis, MLNR (0.50–1.00) is a significant independent prognostic factor for CSS (hazard ratio 2.161, 95% confidence interval 1.327–3.519, *p*=0.002). MLNR (0.50–1.00) has a greater prognostic impact on CSS in female, non-Hispanic white, T3/4, N1b and M1 patients. The lymph node yield (LNY) influences the effect of MLNR on CSS; LNY ≥9 results in MLNR (0.50–1.00) having a higher HR for CSS than MLNR (0.00-0.49). In conclusion, higher MLNRs predict poorer survival in MTC patients. Eradication of involved nodes ensures accurate staging and maximizes the ability of MLNR to predict prognosis.

## INTRODUCTION

Over the past 30 years, there has been a significant increase in the incidence of thyroid cancer (TC) worldwide [1]; it was the fifth most commonly diagnosed types of cancer among women in the United States in 2014 [2]. The prognosis for TC is known to be excellent. However, medullary thyroid carcinoma (MTC), which comprises 5% to 10% of all TCs and occurs as a sporadic or a familial disease, tends to be more aggressive than other TCs (papillary thyroid cancer, follicular thyroid cancer, and so on), frequently having metastasized to cervical lymph nodes (LNs) by the time of diagnosis [3–5]. Surgical management has been demonstrated to be effective in achieving biological recovery and managing metastatic or recurrent disease in some patients with this rare endocrine tumor, which secretes calcitonin. Thorough surgical extirpation of tumor has been the mainstay of treatment for primary MTC; in contrast, the management of the regional lymph nodes has been more controversial. It is now clear that systematic lymph node clearance is optimal for all patients with MTC, the initial treatment of which should include a central neck lymphadenectomy of level VI nodes and total thyroidectomy (TT). Current experience and related single center studies suggest that modified radical neck dissection on the side of clinically palpable nodes is appropriate [6, 7]; however, some authors advocate performing a unilateral or bilateral elective modified radical neck dissection in selected situations [8]. Because of the low incidence of MTC and the consequent lack of prospective studies evaluating its prognosis, there is little convincing evidence concerning the impact of particular procedures on long-term outcomes. However, it has been established that pathologically confirmed lymph node metastasis (LNM) is a major contributor to the stratification of postoperative patients regarding risk of cancer-specific death: the number of LNs harboring metastases has been validated to be an independent prognostic factor in differentiated thyroid cancer (DTC). Unfortunately, this variable depends on the radiographically evident extent as indication for lymph node dissection (LND), has not yet been standardized [9]. It has recently been proposed that the metastatic lymph node ratio (MLNR, ratio between number of metastatic nodes and total number of lymph nodes recovered) is a more reliable and accurate means of stratifying risk than existing staging systems; the MLNR is an important prognostic factor in DTC [10], breast, colorectal [11], and gastric cancers [12].

The concept of MLNR is based on the assumption that it indirectly reflects the extent or stage of the cancer, a higher ratio implying a more advanced cancer stage. However, the evaluation of MLNR in patients with MTC has not been fully studied and there is no consensus on the optimal cutoff for risk categories regarding cancer-specific mortality [13, 14]. The primary purpose of this study was to determine the predictive value of MLNR in SEER (Surveillance, Epidemiology and End Results)-registered MTC patients treated surgically. Second, we used the X-tile program [15] to investigate the cutoff points for the MLNR in stratifying risk of mortality and evaluate whether stratified MLNR complements current nodal classification in patients with MTC. Our final aim was to use the results of our analyses to provide evidence for selection of appropriate treatment strategies for patients with MTC with LNM.

## RESULTS

### Baseline characteristics and identification of MLNR cutoff points

The 890 eligible patients identified in the 20-year study period comprised 375 males and 515 females with a median (range) age of 50 (8–92) years. All patients had undergone lymph node dissection (LND) and 837 (94.0%) TT. According to the final pathology, 135 patients (15.2%) had central LNM and 359 (40.3%) lateral LNM metastases; the LNs were negative in the remaining 396 cases (44.5%). The median (range) of lymph node yield (LNY) was 15 (1–90) and the number of LNs harboring metastases 1 (0–73). The clinicopathologic characteristics of all patients are summarized in Table 1.

**Table 1:** Clinicopathological Characteristics of 890 Patients with MTC Obtained from SEER Database

Variables	Total (%)
Male Sex	375 (42.1)
Age	49.8±16.3 (8–92)
Race African American non-Hispanic white Other ^1^	71 (8.0) 771 (86.6) 48 (5.4)
Treatment TT or near TT Radioisotopes Beam radiation	837 (94.0) 28 (3.1) 130 (14.6)
T stage T1/T2 T3/T4	546 (61.3) 344 (38.7)
N stage N0 N1a N1b	396 (44.5) 135 (15.2) 359 (40.3)
Distant metastasis	119 (13.4)
TNM stage I II III IV	409 (46.0) 102 (11.5) 93 (10.4) 286 (32.1)
Number of positive LNs	6.1±10.3 (0-73)
Number of LNY	24.0±24.1 (1-90)
MLNR	0.25±0.32 (0.00-1.00)

Data are presented as n (%) or mean ± standard deviation (range).

1 including American Indian/AK Native, Asian/Pacific Islander, and Hispanic.

Abbreviations: MTC, medullary thyroid cancer; TT, total thyroidectomy; LNs, lymph nodes; MLNR, metastatic lymph node ratio; LNY, lymph node yield.

**Figure 1:**
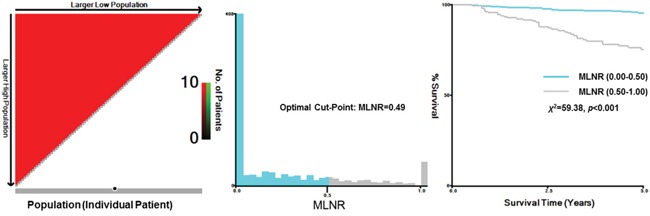
X-tile analysis of survival data from the SEER registry X-tile analysis was done on patient data from the SEER registry, equally divided into training and validation sets. X-tile plots of training sets are shown in the left panels, with plots of matched validation sets shown in the smaller inset. The optimal cut-point highlighted by the black circle in the left panels is shown on a histogram of the entire cohort (middle panels) and a Kaplan-Meier plot (right panels). *P* values were determined by using the cut-point defined in the training set and applying it to the validation set. Figures show MLNR divided at the optimal cut-point (0.49, χ^2^=59.38, *p*<0.001).

The mean duration of follow-up was 50 months (range 6–262 months) and the overall CSS 90.7% at 5-years and 81.7% at 10-years in this cohort of subjects with MTC. X-tile plots were constructed and the maximum of χ^2^ log-rank values of 59.38 produced, applying 0.50 as the cutoff value for dividing the cohort into high and low subsets in terms of CSS (Figure 1).

### Prediction of the MLNR on CSS in patients with MTC

**Figure 2:**
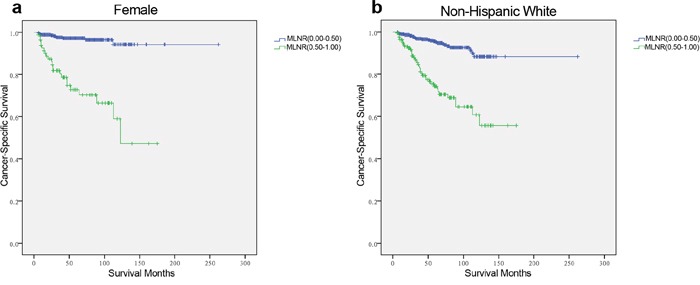
Log-rank tests of cancer-specific survival comparing those who had metastatic lymph node ratio (MLNR) ranged 0.50–1.00 with those who had MLNR ranged 0.00–0.49 for a. female: χ2 = 64.772, *p*=0.001; b. non-Hispanic White: χ2 = 59.502, *p*=0.001

**Figure 3:**
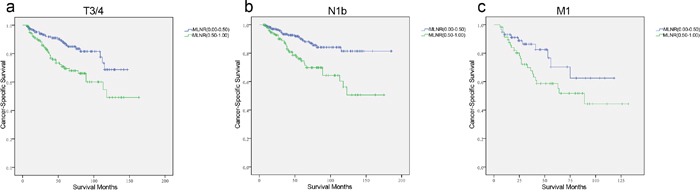
Log-rank tests of cancer-specific survival comparing those who had metastatic lymph node ratio (MLNR) ranged 0.50–1.00 with those who had MLNR ranged 0.00–0.49 for a. T3/4: χ2 = 10.749, *p*=0.001; b. N1b: χ2 = 13.643, *p*=0.001; c. M1: χ2 = 33.195, *p*=0.001

First, the MLNR count was treated as a continuous variable and validated as a significant prognostic factor by univariate log-rank test (χ^2^ = 41.05, *p*<0.001). Next, all patients were classified into the two risk groups according to MLNR with the calculated-cutoff value from X-tile as follows: L1 (MLNR, 0.00–0.49) and L2 (MLNR, 0.50–1.00). Multivariate Cox regression analysis indicated that age, race, T stage, N stage, distant metastasis, and MLNR had significant associations with CSS (Table 2). In particular, patients with a high MLNR had a significantly greater risk of death than those with reference range MLNR (0.00–0.49) (HR 2.161, 95% CI 1.327–3.519, *p*=0.002). To assess the influence of different ranges of MLNR on CSS in strata of patients, the patients were further divided into subgroups according to the variables identified by multivariate analysis. Patients without cervical LNM were excluded from the analysis of stratification by N stage. It was found that L2 (MLNR, 0.50–1.00) was consistently associated with a shorter CSS by Cox multivariate regression in female patients (Figure 2, HR 4.370, 95% CI 2.142–8.915, *p*=0.001), whites (Figure 2, HR 2.762, 95% CI 1.694–4.503, *p*=0.001), T3/4 (Figure 3, HR 2.161, 95% CI 1.267–3.687, *p*=0.005), N1b (Figure 3, HR 2.130, 95% CI 1.222–3.715, *p*=0.008), and M1 subgroups (Figure 3, HR 2.693, 95% CI 1.282–5.656, *p*=0.009) by multivariate analyses. (Table 3)

**Table 2:** Multivariate Cox Regression for CSS in Patients with MTC Obtained from SEER database

Independent variable	Multivariate	
	HR (95% CI)	*P* value
Sex (Female *vs.* Male)	1.239 (0.778-1.974)	0.367
Age (≥45 *vs.* <45)	1.049 (1.031-1.067)	0.001
Race (White vs. Black)	0.422 (0.207-0.860)	0.018
T stage (T3/4 *vs.* T1/2)	3.957 (2.126-7.364)	0.001
N stage N0 N1a N1b	1 (Reference) 2.449 (0.998-6.012) 2.834 (1.063-7.560)	0.049 0.037
Distant metastasis	3.680 (2.275-5.950)	0.001
MLNR (L2 *vs.* L1)	2.161 (1.327-3.519)	0.002

1 including American Indian/AK Native, Asian/Pacific Islander, and Hispanic.

Abbreviations: MTC, medullary thyroid cancer; TT, total thyroidectomy; LNs, lymph nodes; MLNR, metastatic lymph node ratio; CSS, cancer-specific survival; HR, hazard ratio; CI, confidence interval.

**Table 3:** Multivariate Analysis of Effect of MLNR on CSS in MTC According to Clinicopathologic Variables

Variable for subgroups	Multivariate Analysis		
	HR for MLNR (L2 *vs.* L1)	95% CI	*P* value
***Sex***^1^			
Male	1.692	0.922-3.105	0.090
Female	4.370	2.142-8.915	0.001
***Age***^2^			
<45	11.313	2.235-57.259	0.003
≥45	2.153	1.328-3.490	0.002
***Race***^3^			
Black	0.816	0.146-4.560	0.816
White	2.762	1.694-4.503	0.001
***T stage***^4^			
T1/2	2.776	0.865-8.911	0.086
T3/4	2.161	1.267-3.687	0.005
***N stage***^5^			
N1a	3.033	0.909-10.123	0.071
N1b	2.130	1.222-3.715	0.008
***M stage***^6^			
M0	1.703	0.902-3.217	0.101
M1	2.693	1.282-5.656	0.009

1 *P*-value for HR was adjusted for age, race, and TNM stage as covariates.

2 *P*-value for HR was adjusted for sex, race, and TNM stage as covariates.

3 *P*-value for HR was adjusted for sex, age, and TNM stage as covariates.

4 *P*-value for HR was adjusted for sex, age, race, N stage, and M stage as covariates.

5 *P*-value for HR was adjusted for sex, age, race, T stage, and M stage as covariates.

6 *P*-value for HR was adjusted for sex, age, race, T stage, and N stage as covariates.

Abbreviations: MTC, medullary thyroid cancer; MLNR, metastatic lymph node ratio; CSS, cancer-specific survival; HR, hazard ratio; CI, confidence interval.

### Impact of the MLNR on CSS in MTC according to LNY

The importance of the number of LNY has been reported by previous studies that investigated adequacy of staging based on the hypothesis that the fewer lymph nodes recovered, the higher the probability of leaving metastatic nodal disease unresected. Such unresected disease might contribute not only to local recurrence, but decreased CSS [16, 17]. However, according to the present analysis, a higher LNY was not significantly associated with improved CSS in patients with MTC (χ^2^ = 74.324, *p*=0.117; HR 0.962, 95% CI 0.886–1.044). Despite the finding that there is a finite limit to prediction of a favorable prognosis by higher LNY values, it is possible that the LNY does influence the effects of the MLNR. As showed in supplementary Figure 1 (Figure S1), the number of LNY was unevenly distributed between one and 90. The upper and lower quartiles of the LNY counts were therefore calculated to divide patients into subgroups to assess the influence of different LNY ranges on the effects of MLNR (Table 4). First, the HR for MLNR on CSS increased with increasing LNY, indicating that achieving a greater LNY enhances the predictive value of the MLNR. Second, when the LNY exceeded 15, the HR for MLNR on CSS decreased, even though the MLNR had a consistently significant predictive effect on CSS. It is thus likely that the influence of MLNR on survival is similar for all values of LNY above the range 6–15. Therefore, we further analyzed individual LNY counts from 6 to 15 (Table 5). In patients with an LNY count nine or more, those with higher MLNRs (0.50–1.00) had higher HR (HR 15.388, 95% CI 6.525–36.287, *p*=0.001) for cancer-specific death than those within the reference MLNR range (0.00–0.49). Thus, our results indicate that the MLNR reaches its maximal significance as a potential predictor of survival of patients with MTC at LNY ≥9.

**Table 4:** Multivariate Analysis of Effect of MLNR on CSS in MTC According to LNY

LNY^*^	Number of patients	Multivariate Analysis		
		HR for MLNR (L2 *vs.* L1)	95% CI	*P* value
1-5	256	7.570	2.956-19.389	0.001
6-15	200	14.958	4.201-53.254	0.001
16-37	220	9.978	1.266-78.638	0.029
38-90	214	8.761	2.197-34.943	0.002

* The cutoff points for the number of LNY were calculated from the quartiles of individual LNY counts.

*P*-value for HR was adjusted for sex, age, race, T stage, and N stage and M stage as covariates.

Abbreviations: MTC, medullary thyroid cancer; MLNR, metastatic lymph node ratio; LNY, lymph node yield; CSS, cancer-specific survival; HR, hazard ratio; CI, confidence interval.

**Table 5:** The Impact of MLNR on CSS in MTC According to LNY from 6–15

LNY*	Number of patients	Multivariate Analysis		
		HR for MLNR (L2 *vs.* L1)	95% CI	*P* value
≥6	634	11.472	5.361-24.547	0.001
≥7	599	13.980	6.215-31.443	0.001
≥8	572	15.129	6.655-34.389	0.001
≥9	548	15.388	6.525-36.287	0.001
≥10	532	14.735	6.236-34.810	0.001
≥11	509	13.646	5.746-32.409	0.001
≥12	500	13.340	5.606-31.739	0.001
≥13	481	14.089	6.034-37.734	0.001
≥14	462	13.317	5.194-34.141	0.001
≥15	450	10.543	3.937-28.233	0.001

Abbreviations: MTC, medullary thyroid cancer; MLNR, metastatic lymph node ratio; LNY, lymph node yield; CSS, cancer-specific survival; HR, hazard ratio; CI, confidence interval.

## DISCUSSION

Because regional involvement of LNs an important prognostic factor in patients with MTC, LN assessment is fundamental to pathological staging in such patients. However, there is currently no consensus regarding the appropriate extent of LND in these patients, likely because the significance of extent of LND or LN number on prognosis has not yet been determined. MLNR carries prognostic significance in several cancers including DTC, implying that it reflects the completeness of eradication of local LNs and the burden of regional nodal disease. The MLNR is likely a function of the total number of LNY. In our large population-based study, we found that the MLNR is an independent prognosis factor in patients with MTC, especially in those with locally advanced (T3/4, N1b) disease and distant metastases. The LNY had a weaker correlation with CSS than the MLNR; however, interestingly, the influence of MLNR on prognosis varied slightly with range of LNY count. Our data indicate that an LNY count ≥9 maximizes the ability of the MLNR to predict a poor CSS in patients with MTC.

MTC, a relatively rare type of thyroid malignancy, is associated with poorer survival than papillary or follicular TC. Our cohort had a 81.7% CSS at 10-years, which is similar to those of previous studies, which have that reported 10-year survival rates ranging from 69% to 89% [5, 18]. The current TNM staging classification takes into account tumor size, invasion, and evidence of regional lymph node or distant metastases as risk factors when stratifying the risk of cancer death in an individual with MTC postoperatively. In addition, Machens et al. have reported that metastases in more than 10 lymph nodes and involvement of more than two lymph node compartments correlate strongly with survival in patients with MTC [17]. We found a significant correlation between the MLNR and CSS by Kaplan–Meier analysis; further, X-tile plots calculated a cutoff value of 0.50 when the MLNR was used to define high or low-risk subsets using CSS as a binary variable. In a Cox proportional hazards model adjusted for age, race, and TNM stage as covariates, a binary MLNR still significantly influenced the CSS. Several clinical prognostic factors have previously been identified for MTC, including age (≥45 years), tumor stage (T3/4), and the presence of lymph node (N1) and distant metastases (M1) [19, 20]: the current analyses proved all of these to be significant predictors of CSS. In particular, in the analyses of subgroup stratified by the above factors, a higher MLNR (0.50–1.00) was found by multivariate analyses to be associated with lower CSS in female and white subjects and those with T3/4, N1b, or M1 disease. This indicates that the appropriateness of LND and the usefulness of MLNR are clinically more significant for tailoring treatment and follow-up protocols in patients with MTC and the conventionally recognized risk factors.

It is known that identification of more LNs in surgical specimens indicates more complete excision of tumors and their draining nodes in patients with MTC. The fact that increasing LNY was not associated with improved survival according to continuous variable analysis suggests that the benefit to increasing the number of LNY is finite and largely dominated by the effects of other factors such as patient age, primary tumor stage, distant metastasis, and so on. Because the LNY as a continuous factor is not a significant predictor of prognosis, we considered investigating a cutoff value for further analysis by X-tile plots inappropriate. We thus used the upper and lower quartiles to test the influence of LNY counts on MLNR in risk stratification for cancer death. Beyond the cutoff limit of nine, further increases in LNY count did not improve the HR of a higher MLNR for CSS; we therefore identified nine as the potential cutoff value for LNY. The LNY may be an indicator of quality of surgical care or pathology; achieving certain LNY counts, such as nine according to the present study, may minimize the chance of micro-metastases remaining within regional LNs and enable the MLNR to reach its maximal prediction for CSS (HR 15.388, *p*=0.001) [16]. In addition, the chance of under-staging is low and the value of MLNR for prognosis more apparent. One important case to point out would be that a central LND alone (to define N1a disease) would possibly draw a LNY of 9; under this circumstance, it could be cautious of the LNY prediction for CSS, due to the subgroup analysis that MLNR was not associated with CSS significantly within N1a range (Table 3).

The surgical treatment of MTC is influenced by the following considerations: (1) the clinical course of MTC seems to be more aggressive than that of DTC, with higher mortality rates; (2) MTC arising from the parafollicular or C-cells does not take up radioactive iodine; (3) hormone suppression is not indicated because MTC has a neuroendocrine origin; and (4) nodal metastases are present in more than 70% of patients at diagnosis. Recently published guidelines for management of MTC will be useful to clinicians treating these patients. Preoperative ultrasonographic and computed tomography evaluation of neck nodes is helpful for acquiring a detailed evaluation of nodal architecture, allowing for easier perioperative localization, biopsy, and marking of cervical nodes. In patients with palpable MTC, central LND reportedly achieves better survival rates than those retrospectively achieved with procedures involving removal of grossly involved nodes only [21]. The recommended treatment for the primary tumor at initial surgery is therefore TT accompanied by central LND [22]. For lateral compartment involvement, the rates of LNM reportedly range from 47% to 75%. Therefore, preoperative imaging or intraoperative assessment suggesting lateral LNM is useful in delineating the extent of nodal spread and in planning the extent of LND. Consideration should be given to performing a prophylactic ipsilateral or bilateral LND with clinically apparent or palpable MTC with the aim of achieving long-term control and cure. Of note in this series, higher MLNRs in patients with conventional risk factors, such as locally advanced stage or distant metastasis, were more significant predictors of mortality. Therefore, in female patients, those age ≥45 years, and those with T3/4, and M1 disease, a systematic (compartment-oriented) approach to the removal of all nodal tissue in the lateral neck is important not only for complete removal of local metastases, but also for achieving more LNY to allow better staging and improve the ability of MLNR to predict prognosis. Although in MTC patient with M1 disease, the regionally dissection weighs less important than systemic treatment; the comprehensive treatment, such as target therapy, could not maximize effectiveness for M1 disease until the complete excisions for primary tumor and regionally metastatic lymph nodes. We thus suggest a complete and safe to obtain a radical treatment for neck nodes and avoid potential unneeded local morbidity in MTC patients with distant metastasis.

This large population-based study evaluating the effect of MLNR on prognosis in patients with MTC has several potential limitations. First, MTC occurs in hereditary (25% of cases) and sporadic (75%) forms. Sporadic MTC tends to be more aggressive than hereditary MTCs, and the former frequently metastasize to cervical LNs; thus, the predictive value of MLNR may differ between these two types of MTC. Second, although the number of LNY and positive LNs can easily be obtained from the SEER database, we still excluded some cases because of insufficient information regarding N stage and number of LNs harboring metastases. The quality of LND and quantity of LNY and LNs harboring metastases examined by the pathologist do substantially influence outcomes. Third, the SEER database does not provide information about calcitonin, which is produced by thyroid C-cells and MTC cells and is helpful for screening patients at risk of MTC and follow-up of patients who have undergone treatment for MTC. Following primary surgery for MTC, persistent or recurrent increases in calcitonin concentrations indicate the presence of local, regional, or distant disease. Thus, our analyses could not adjust for these potential confounding factors.

In conclusion, our analysis of the SEER database revealed a significant association between MLNR and CSS in patients with MTC who had undergone surgical treatments for LNM. Higher MLNR is predictive of poorer CSS, and the impact of MLNR on MTC survival is more significant in female and non-Hispanic white patients and in those with T3/4, N1b and M1 disease. Although the number of LNY does not predict overall survival in MTC, it does influence the ability of the MLNR to predict prognosis. Systematic dissection of cervical nodal tissue provides better staging and application of the MLNR as a predictor of prognosis in patients with MTC.

## MATERIALS AND METHODS

### Selection of patients from the SEER database

For this study, data were extracted from the SEER cancer registry, which is a population-based registry sponsored by the National Cancer Institute that collects information on cancer incidence and survival from 17 population-based cancer registries, including approximately 28% of the US population [23]. SEER data contain no identifiers and are publicly available for studies on cancer-based epidemiology and health policy. The National Cancer Institute's SEER*Stat software (Surveillance Research Program, National Cancer Institute SEER*Stat software, www.seer.cancer.gov/seerstat) (Version 8.1.2) was used to identify patients who had been diagnosed with a single primary MTC from 1991 to 2012. Patients who had undergone thyroidectomy (lobectomy, near TT and TT), lymph node dissection (LND, LNY≥1) and postoperative therapy for MTC were included. Histology types were limited to MTC (8510, representing the histology of MTC in SEER coding manuals). Exclusion criteria were as follows: insufficient data concerning number of LNs, unknown clinicopathologic-profile, short duration of follow-up (<6 months), undetermined histology or other type of thyroid cancer (DTC, anaplastic thyroid cancer, and so on).

### Ethics statement

This study was performed in compliance with the Helsinki Declaration, approved by an independent ethics committee/institutional review board at Fudan University Shanghai Cancer Center and conducted in accordance with the approved guidelines. Data released from the SEER database does not require informed patient consent because it contains no identifiers and is publicly available. We obtained permission to access the research data file in the SEER program from the National Cancer Institute, USA (reference number 10817-Nov2013).

### Assessment of clinicopathological variables

Age, sex, race, surgical procedures, adjuvant therapies, TNM stage, number of metastatic LNs, number of lymph nodes recovered (represented by LNY), and survival time were extracted from the SEER database. Race was categorized as African American, non-Hispanic white, and others (American Indian/AK Native, Asian/Pacific Islander, and Hispanic) as provided by the SEER database. The MLNR was calculated as the number of positive regional nodes divided by the number of regional nodes from the cervical compartment examined. The 2010 TNM classification of American Joint Committee on Cancer/International Union Against Cancer (AJCC/UICC) was used [24]. The endpoint of the present study was the cancer-specific survival (CSS), which was calculated from the date of diagnosis to the date of cancer-specific death and is shown as “SEER cause-specific survival” in the SEER database.

### Statistical analysis

Survival rates were generated using a Kaplan–Meier curve and differences were compared with the log-rank test. MLNR cutoff points were analyzed using the X-tile program (http://www.tissuearray.org/rimmlab/), which identified the cutoff with the minimum *p* values from log-rank χ^2^ statistics for the categorical MLNR in terms of CSS [15]. A Cox proportional hazards regression model was built to evaluate the effects of variables on cancer mortality in MTC patients and then adjusted by multivariate analysis for patient characteristics (as listed above) and tumor characteristics. The hazard ratios (HRs) for relationships between each variable and cancer-specific mortality were calculated using a binary Cox regression model. All confidence intervals (CIs) are presented at the 95% confidence level. All *p* values are two-sided. *P*<0.05 was considered statistically significant. Statistical analysis was performed using SPSS software, version 13.0 (SPSS, Chicago, IL, USA).

## SUPPLEMENTARY MATERIALS FIGURE


