# Supplementary material for: Metastatic lymph node ratio can further stratify risk for mortality in medullary thyroid cancer patients: A population-based analysis
Source: Oncotarget. 2016 Aug 31;7(40):65937–45. doi: 10.18632/oncotarget.11725 (PMC5323204; doi:10.18632/oncotarget.11725)
Supplement: Supplementary file 1 [file oncotarget-07-65937-s001.pdf]

Metastatic lymph node ratio can further stratify risk for mortality in medullary thyroid cancer patients: A population-based analysis

SUPPLEMENTARY FIGURE

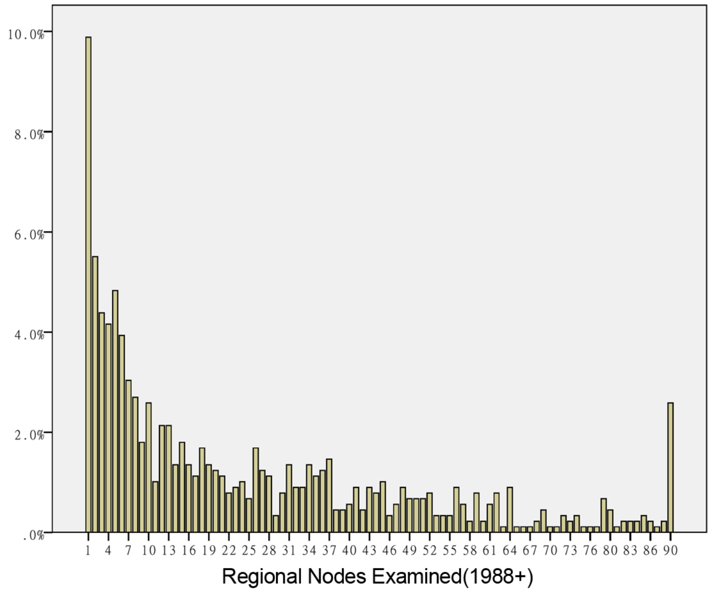

Supplementary Figure S1: The distribution for the number of lymph node yield (LNY).
